# Cost-saving Minimal Incision Endoscopic-assisted Cubital Tunnel Release Using Simple Surgical Instruments: Case Series

**DOI:** 10.7759/cureus.5914

**Published:** 2019-10-15

**Authors:** Woraphon Jaroenporn, Pradit Predeeprompan, Jaruwat Vechasilp, Torpon Vathana, Roongsak Limthongthang

**Affiliations:** 1 Hand and Microsurgery, Orthopaedic Surgery, Police General Hospital, Bangkok, THA; 2 Orthopaedics, Police General Hospital, Bangkok, THA; 3 Orthopaedic Surgery, Siriraj Hospital, Bangkok, THA

**Keywords:** decompression, compressive neuropathy, cubital tunnel syndrome, minimal invasive, endoscopic, in situ decompression, cost effective

## Abstract

Cubital tunnel syndrome (CuTS) is a well-recognized compressive neuropathy worldwide. With technological advancement, endoscopy is introduced to facilitate the procedure. However, there are concerns about the excessive cost that comes with special instruments. This article aims to provide the results of the cost-saving endoscopic-assisted cubital tunnel release surgical technique that uses the normally available operating instruments.

A retrospective review was performed of the nine patients that were diagnosed with CuTS and underwent minimal incision endoscopic-assisted cubital tunnel release in Police General Hospital. Patients were followed up to sixth month postoperation. The modified McGowan classification was used to determine the severity of symptoms. Surgical outcomes were evaluated by the modified Bishop classification, visual analog score (VAS), and patients' satisfaction. Other factors investigated were scar pain and peri-incisional numbness and hematomas.

The incisions were measured as 7-9 mm. All patients reported having a pain score of 1 on the third day. Seven of nine patients were able to return to work one day after surgery. Modified Bishop score showed five excellence, three good, and one fair after two weeks. There was no surgical-related complication found. All patients noted the excellence satisfaction of the procedure.

The minimal incision endoscopic-assisted cubital tunnel release has shown favorable outcomes with the cost-saving of simple instruments. However, a large prospective trial may be needed for further study.

## Introduction

Cubital tunnel syndrome (CuTS) is the second most common peripheral neuropathy [1]. There are three concepts in surgical approaches for the treatment of CuTS, including in situ decompression, transposition, and epicondylectomy [2]. Recently, simple decompression has steadily gained support for its better cost-effectiveness and lower complication rate [3-4]. There are many variations in the open in situ cubital tunnel release technique from the classical to the minimal incision. The classical incision normally uses an 8-10 cm length of incision, which allows wide exposure but endangers the median antebrachial cutaneous nerve (MABC) as well as causes postoperative pain and increases healing time [2,5]. Various minimal incision techniques were proposed using the mobility of the soft tissue around the elbow to shorten the recovery time and reduce scar tenderness and iatrogenic injury to MABC [6]. The cadaveric studies show that the compressive structures spanning from an average of 8.2 cm. proximal to 6.4 cm distal from the medial epicondyle [7], Josep Said et al. stated that a 4-cm open incision is needed to allow the visualization of 9 cm [8]. There are many studies that support that endoscopic cubital tunnel decompression has a smaller incision, greater field of view, better short-term outcome, and fewer complications [9]. Many authors have used various special instruments that raise concern on excessive expense and cost-effectiveness [5]. To our knowledge, few studies used basic operating instruments, with a 1.5-2 cm incision, which is not too different from minimal incision open cubital tunnel release. This retrospective case series was performed to propose the results of the novel procedure of the 7-9 mm incision, which is easily replicable and cost-saving.

## Case presentation

Materials and methods

A retrospective review was performed to identify patients who were diagnosed with CuTS between August 2017 and August 2019 and underwent minimal incision endoscopic-assisted cubital tunnel release performed by a single surgeon. Nine patients were identified. The severity of the symptoms was classified with the modified McGowan classification (Table 1) [10]. Radiographs of all nine elbows showed no deformity or significant osteoarthritis. In the modified Bishop classification (Table 2) [11], the visual analog score, patients' satisfaction were used to evaluate the patients in the first 10 days postoperation via telephone by the same resident who was not involved in the study. The patients were appointed to the clinic in the second, fourth, and sixth weeks and then in the third and sixth months with the same evaluation protocol.

**Table 1:** Modified McGowan score

Grade	Description
1	Purely subjective symptoms causing dysfunction in daily activities
2A	Muscle weakness with or without subjective symptoms, without detectable atrophy
2B	Muscle weakness with or without subjective symptoms, with detectable atrophy
3	Disabling weakness, marked intrinsic atrophy, and profound sensory disturbances

**Table 2:** Modified Bishop score

Items	Score
Residual symptoms	
None	3
Little/Intermitted	2
Moderate	1
Severe	0
Subjective improvement	
Better	2
Unchanged	1
Worse	0
Ability to work	
Working in old job	2
Changed job due to complains	1
Incapable of working	0
Muscle strength	
Better	1
Unchanged	0
Evaluation	
Excellent	8-9
Good	6-7
Fair	4-5
Poor	<3

Surgical technique

**Figure 1:**
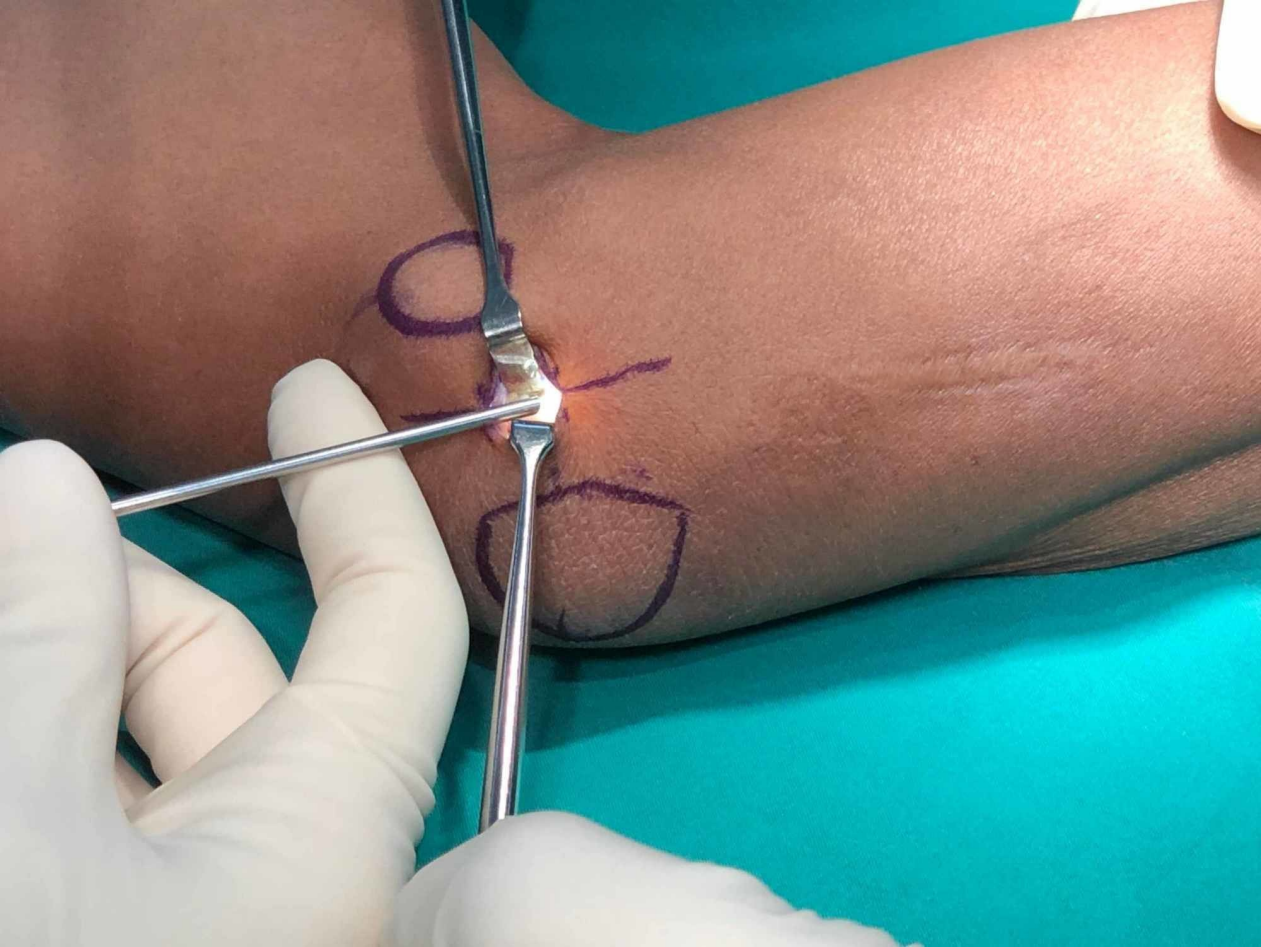
The ulnar nerve is identified with a 7-9 mm incision using Senn retractors to facilitate the mobility of the soft tissue around the elbow

**Figure 2:**
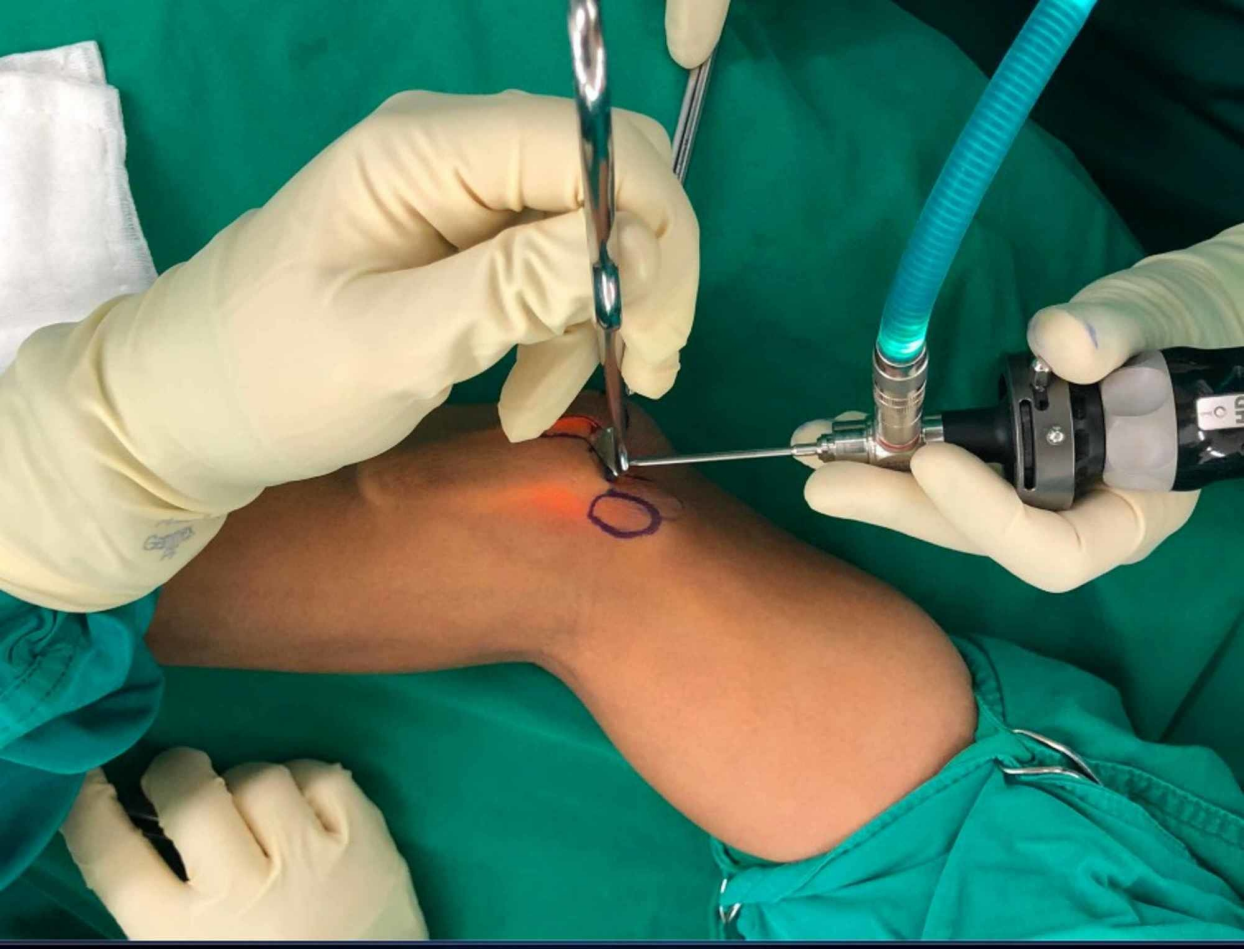
The 2.5 mm endoscope is utilized to illuminate and magnify the surgical area

**Figure 3:**
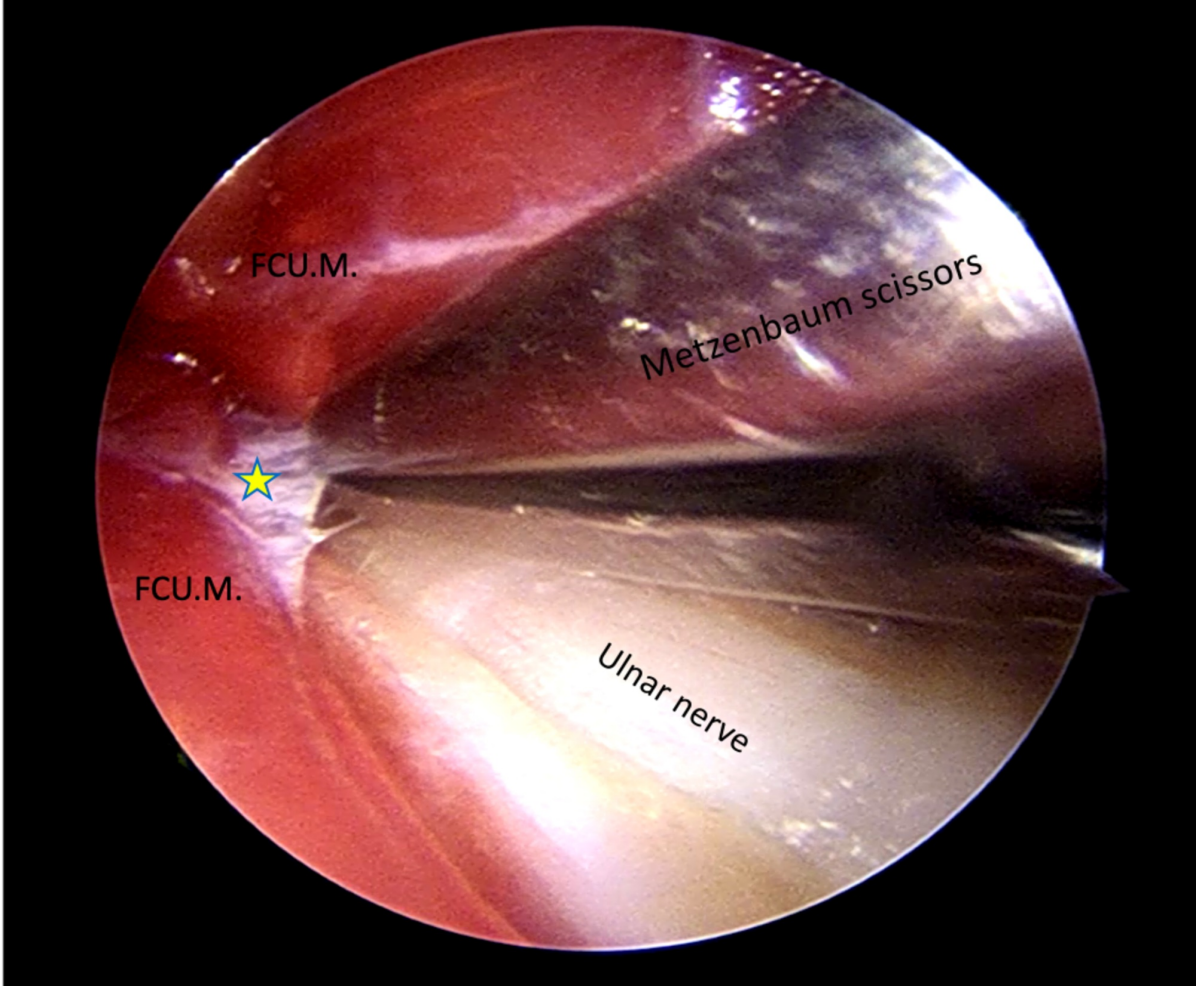
Endoscopic view: a narrow Metzenbaum scissors is introduced to dissect the fascia between the two heads of the flexor carpi ulnaris muscle FCU.M.: Flexor carpi ulnaris muscle; star: facia between the two heads of the flexor carpi ulnaris muscle

The operation is performed under general anesthesia with a pneumatic tourniquet. The affected arm is positioned on an arm board set perpendicular to the bed. The sterile technique is performing in the standard fashion. The medial epicondyle, olecranon, along with the ulnar nerve, are identified and marked. The longitudinal, 7 to 9-mm long incision is made on the skin overlying the ulnar nerve. The Senn retractor is used to gain better visualization (Figure 1). Metzenbaum scissors are used for dissecting soft tissue while taking care not to injure the MABC. After incising the aponeurosis and fascia compressing the ulnar nerve, the small Langenbeck retractor is introduced along the length of the incision and then rotated 90° in order to elevate the soft tissue above the ulnar nerve. The 2.5 mm, 30° endoscope is now introduced into the field (Figure 2). After the ulnar nerve is localized, the curved short blade Metzenbaum is now used to cautiously dissect the compressing soft tissue overlying the ulnar nerve under a clear endoscopic view (Figure 3). Nine cm. proximal, as well as distal, to the medial epicondyle of the ulnar nerve is decompressed with the same technique. Care is taken to avoid injury to the branches and vessels of the ulnar nerve. After satisfaction, meticulous hemostasis is obtained. The surgical wound is closed with a single, absorbable, 5-0 monofilament suture. The patients are instructed to start motion immediately after the operation.

Results

There were nine patients with CuTS, including five males and four females, with an average age of 49 years. One patient presented with Grade 1 severity according to the McGowan classification. four presented with Grade 2a, and the other four with Grade 2b (Table 3). Postoperative follow-up of the first 10 days was performed by a telephonic questionnaire and the wound was evaluated by photos through e-mail. There was no complication-related surgery in all nine patients, such as for infection, new or different paresthesia, peri-incisional numbness, hematomas, or dehiscence. There was no ulnar nerve subluxation prior to and after the operation. The length of the surgical incision was 7-9 mm. The visual analog score shows that on the first day after surgery, six patients reported a scale of 1, two patients reported 2, and one patient experienced a scale of 3 (Table 4). All patients had no pain in the second week postoperation. Only two patients needed rest because of concern for their wound healing but not the pain while the others were able to return to work one day after the operation. Modified Bishop score showed an excellent result for four patients in the first week, three good results, and two fair (Table 5). In three months, the results were five excellence and four good. In six months, all patients reported an excellent result. All patients reported excellent satisfaction both in the first week and the sixth month, which they would undergo the same operation again given the opportunity.

**Table 3:** Demographic data

Patient	Sex	Age	Diagnosis	Modified McGowan score	Duration of symptoms (months)
No.1	F	48	Rt. CuTS	2b	10
No.2	M	59	Lt. CuTS	2a	9.5
No.3	F	54	Lt. CuTS	2a	22
No.4	M	47	Rt. CuTS	2b	8
No.5	M	50	Rt. CuTS	2b	12
No.6	F	52	Rt. CuTS	2a	13.4
No.7	M	42	Rt. CuTS	2a	27
No.8	M	46	Lt. CuTS	2b	7
No.9	F	41	Rt. CuTS	1	6

**Table 4:** Postoperative pain score

Postoperative pain score (VAS)															
Patient	Day(s)										Week(s)			Month(s)	
	1	2	3	4	5	6	7	8	9	10	2	4	6	3	6
No.1	1	1	1	1	1	0	1	0	1	0	0	0	0	0	0
No.2	1	1	1	1	1	1	1	1	1	0	0	0	0	0	0
No.3	1	2	1	1	1	1	2	1	0	0	0	0	0	0	0
No.4	1	1	1	1	1	1	0	0	0	0	0	0	0	0	0
No.5	2	1	1	1	1	2	1	1	1	0	0	0	0	0	0
No.6	1	1	1	1	1	1	1	1	0	0	0	0	0	0	0
No.7	1	1	1	1	1	1	0	0	0	0	0	0	0	0	0
No.8	1	2	1	1	1	1	1	1	1	0	0	0	0	0	0
No.9	3	2	1	1	0	0	0	0	0	0	0	0	0	0	0

**Table 5:** Postoperative modified Bishop score

Modified Bishop score				
Patient	Week(s)		Month(s)	
	1	2	3	6
No.1	7	7	7	7
No.2	8	8	8	8
No.3	5	6	6	7
No.4	8	8	8	8
No.5	7	8	8	8
No.6	7	7	7	7
No.7	5	6	6	7
No.8	8	8	8	8
No.9	8	8	8	9

## Discussion

In situ decompression of the ulnar nerve has steadily gained support from many studies for its effectiveness and low complication rate [3]. Historically, open cubital tunnel release has been the standard procedure for most surgeons. There were many common complications, such as peri-incisional pain or numbness, wound dehiscence, hematomas, or iatrogenic nerve injury, reported. These complications could be related to the large incision required [6]. Recently, minimally invasive procedures, such as the minimal incision and endoscopic techniques, have become more favorable, with the theoretical benefits of a smaller incision, reduced pain and time of return to work, and iatrogenic injury to nerve branches, vessels, and surrounding tissue [12]. With the technique that this study proposed, the incision needed was 7-9 mm, which is considered almost half that in the minimal incisional technique [6,13]. But it still has the benefit of the endoscope maintaining clear visualization along the decompression side. All the patients reported a pain score of 1 out of 10 only on Day 3 and were completely pain-free after Day 10. Seven of nine patients could return to work after one day of surgery. The others refused to go back to work because of concern for their wound healing but not the pain. These results may contribute to the smaller incision as compared to previous studies. There were two patients who relatively have fair results from the modified Bishop score, which may be caused by the duration of symptoms before surgery.

The limitations of this study are its retrospective manner and small sample size.

## Conclusions

This study purposed the minimal incision endoscopic in situ ulnar nerve decompression technique as being cost-effective, with favorable outcomes, without the use of special instruments, and easily replicable. A large prospective trial may be useful for further evaluation of the technique.
